# Hierarchical Suppression Based Matched Filter for Hyperspertral Imagery Target Detection

**DOI:** 10.3390/s21010144

**Published:** 2020-12-28

**Authors:** Ce Gao, Yiquan Wu, Xiaohui Hao

**Affiliations:** College of Electronic and Information Engineering, Nanjing University of Aeronautics and Astronautics, Nanjing 211106, China; gaoce1116@nuaa.edu.cn (C.G.); xiaoxiaohuih@nuaa.edu.cn (X.H.)

**Keywords:** hierarchical structure, matched filter, hyperspectral target detection, background suppression

## Abstract

Target detection in hyperspectral imagery (HSI) aims at extracting target components of interest from hundreds of narrow contiguous spectral bands, where the prior target information plays a vital role. However, the limitation of the previous methods is that only single-layer detection is carried out, which is not sufficient to discriminate the target parts from complex background spectra accurately. In this paper, we introduce a hierarchical structure to the traditional algorithm matched filter (MF). Because of the advantages of MF in target separation performance, that is, the background components are suppressed while preserving the targets, the detection result of MF is used to further suppress the background components in a cyclic iterative manner. In each iteration, the average output of the previous iteration is used as a suppression criterion to distinguish these pixels judged as backgrounds in the current iteration. To better stand out the target spectra from the background clutter, HSI spectral input and the given target spectrum are whitened and then used to construct the MF in the current iteration. Finally, we provide the corresponding proofs for the convergence of the output and suppression criterion. Experimental results on three classical hyperspectral datasets confirm that the proposed method performs better than some traditional and recently proposed methods.

## 1. Introduction

Hyperspectral imagery (HSI) is composed of a three-dimensional data structure with spatial-spatial-spectral components, which is viewed as an image cube [1,2]. Typically, the sensor acquires the pixels in the scene in the form of spectral vectors. Hundreds of adjacent spectral bands constitute elements of the vectors, which can be used to distinguish different materials on the basis of their different electromagnetic reflection energy [3,4]. As a result, HSI target detection has huge application prospects, which span military [5,6], agriculture [7,8], prospecting [9], and disaster monitoring fields [3,10,11].

In the past few decades, researchers and scholars have proposed several hyperspectral target detection algorithms. Spectral angle mapper (SAM) [12] is generally considered as the simplest one since it does not require any assumption on data distribution. The constrained energy minimization (CEM) [13,14] minimizes the output energy by constructing a finite impulse response (FIR) filter. Based on CEM, a robust detector uses an inequality constraint on CEM (CEM-IC) to guarantee that the outputs of target spectra are larger than one [15]. Some traditional target detection algorithms are usually based on the statistical information of the target and background spectra [16], such as spectral matched filter (SMF) [3,17], adaptive matched filter (AMF) [18], and adaptive coherence/cosine estimator (ACE) [19,20]. These methods assume that the Gaussian distribution exists on the target and background spectra and adopt the generalized likelihood ratio test (GLRT) [16] to distinguish the target and background pixels. Additionally, the subspace model is applied to describe the difference between the target and background spectra. In this model, the information of the target and background spectra is contained in the target and background subspaces, respectively. By constructing the subspace model, a number of classical target detection algorithms have been developed, such as orthogonal subspace projection (OSP) [21,22], matched subspace detector (MSD) [23], and adaptive subspace detector (ASD) [19,24]. The above subspace-based target detectors extend the dimension of target signatures for overcoming the spectral variability problems [3].

As machine learning methods are introduced to HSI target detection, the classical subspace-based target detectors are also in further development. Instead of exploiting first- and second-order statistics [25], the generalization of kernel-based machine learning extends these HSI target detectors to their corresponding nonlinear (kernel) versions, such as kernel MSD [26], kernel ASD and kernel OSP detectors [4,27]. In addition, sparsity-based target detectors depend on the conception that each pixel in HSI can be represented by as few atoms as possible in a given spectral dictionary. According to the sparse representation of known target signatures, several methods develop into significant target detection strategies such as sparsity-based target detector (STD) [28], simultaneous joint sparsity-based detector [29], a sparse representation-based binary hypothesis (SRBBH) model [30], etc. [31,32,33].

The prior target spectral signature is one key point in hyperspectral target detection. However, the spectral variability is generated in the signal transmission path, which greatly affects the quality of the detection results, including uncompensated errors in the sensor, uncompensated atmospheric and environmental effects [34], etc. In [35], the authors propose an automatic robust iteratively reweighted method to ease the spectral variation. An optimal spectrum is obtained by iterating the target spectrum. In [36], the hyperspectral target detector, constructed by an effective spectral feature combined with the spectral reflectance and spectral derivative, is able to tolerate the spectral variations of the same material.

Recently, detection methods based on the hierarchical structure have been proposed. Rather than directly improving the quality of the target spectrum, their work concentrates on the background suppression through one hierarchical structure in the detection task. The hierarchical CEM (hCEM) [37] detector proposed by Zou et al., consists of different layers of classical CEM detectors. In hCEM, the output of each layer’s spectra is transformed by a nonlinear suppression and then used to update the HSI input for the next iteration. Furthermore, motivated by the spirit of the hierarchical structure, Hao et al., propose an angle distance-based hierarchical background separation method (ADHBS) [38] to enlarge the angle distance between the target and background pixels. Using the ADHBS method, the background spectral vectors gradually move to the direction perpendicular to the target spectral vector.

Since the traditional matched filter (MF) detector only performs single-layer detection on HSI input, it is evident that the background suppression is not sufficient, so the target and the background cannot be completely separated. In this paper, we impose the hierarchical background suppression on the MF detector (HSMF), designed to filter the HSI input several times to strengthen background suppression. In our method, the output of each layer imparts an important effect on the HSI input of the next layer. Since the traditional MF detector has the characteristic of suppressing the background and retaining the target, which is directly reflected in the output of each pixel, we choose the mean value of the output for each layer as a suppression criterion. Compared with hCEM and ADHBS, which also adopt a hierarchical structure, HSMF only needs fewer iterations to complete the output convergence based on this suppression structure. In addition, for hCEM and ADHBS, the whole HSI input will be suppressed in the next iteration, where the background convergence speed is faster than the target pixel, so that the targets can be separated from the background. Our method only suppresses the background, but the target pixels remain unchanged, and the target data are completely preserved in each iteration. The contributions of our work are summarized as follows:

1. Compared with the traditional MF detector, since the hierarchical structure greatly enhances the background suppression, the detection performance of HSMF is significantly improved.
2. The suppression criterion we adopted quickly completes the convergence of the proposed algorithm, so it only takes a few iterations to output the result. In addition, we provide theoretical proofs for the convergences of the suppression criterion and output energy.
3. Comparison experiments on three real hyperspectral images show that the proposed method is superior to the recently proposed algorithms and some classical detectors.

The organization of the rest of this paper is as follows. In Section 2, we briefly introduce the MF detectors and applications of hierarchical structure. In Section 3, our proposed method is explained in detail, and some theoretical analyses are given. Extensive experiments are presented in Section 4. Some discussions are demonstrated in Section 5. Finally, we draw conclusions in Section 6.

## 2. Related Work

### 2.1. Brief Introduction to Matched Filter Detectors

A spectral observation sample consisting of *B* spectral bands can be represented in a vector form as $x={\left[x_{1},x_{2},\ldots ,x_{B}\right]}^{T}$. Typically, the input spectral data are mostly only composed of the background clutter. However, when the spectral signature of targets mixes background clutter noise, the mixed input is equivalent to a linear additive model [3]. The model for MF is given by the following two hypotheses:

(1) $$\begin{array}{l} H_{0}:x=n,\;\mathrm{target}\;\mathrm{absent}\\ H_{1}:x=as+n,\;\mathrm{target}\;\mathrm{present} \end{array}$$

where $s={\left[s_{1},s_{2},\ldots ,s_{B}\right]}^{T}$ is the spectral signature of the desired target with *B* spectra bands, a is the target abundance measure, and **n** is the background clutter noise.

Generally, the MF detector follows a specific assumption: the background clutter covariance matrix and the spectral signature of targets are known in the form of a linear additive model. According to the GLRT [16], the MF output for a test spectral input vector **x** is expressed by

(2) $$y_{\mathrm{MF}}(x)=\frac{s^{T}{\hat{C}}^{-1}x}{s^{T}{\hat{C}}^{-1}s}\overset{H_{1}}{\underset{H_{0}}{≷}}\eta_{\mathrm{MF}}$$

where $\hat{C}$ and **s** represent the estimated covariance matrix and the prior target spectrum, respectively. If the output $y_{\mathrm{MF}}$ is larger than the given threshold $\eta_{\mathrm{MF}}$, the input sample is viewed as a target; otherwise, it is considered as a background pixel.

Instead of using global background clutter statistics, adaptive MF (AMF) [18] designs a dual window construction consisting of two regions. The inner window is framed based on the size of the target, and the outer window consists of the local background clutter, which makes the AMF detector more adaptive to the local background clutter statistics.

Based on the AMF detector, imposing the regularization term constructs a more stable detector [39]. The output function of the regularized AMF is expressed by

(3) $$y=\frac{s^{T}{\left(\hat{C}+\beta I\right)}^{-1}X}{s^{T}{\left(\hat{C}+\beta I\right)}^{-1}s}$$

where *β* is the regularization coefficient, and **I** is the identity matrix. In the regularized AMF, a quadratic penalization term is beneficial to keep the covariance matrix non-singular and make the regularization coefficient tend to zero.

### 2.2. Applications of Hierarchical Structure in Hyperspectral Target Detection

Limited by the single-layer structure, the traditional target detectors cannot completely separate the target and background pixels. Hence, some hyperspectral target detection methods make use of a hierarchical structure on the basis of traditional algorithms. Both hCEM and ADHBS introduce a hierarchical structure to the traditional algorithms to improve their background suppression ability.

In hCEM [37], the authors apply one hierarchical structure to the traditional CEM detector. After each iteration, the magnitude of the background spectra is reduced, while the target spectra are preserved. The hCEM output vector of the *k*th layer is formulated as:

(4) $$y^{k}=\frac{d^{T}R_{k}^{-1}}{d^{T}R_{k}^{-1}d}X^{k}$$

where $X^{k}$ represents the spectra matrix in the *k*th layer, **d** is the prior target spectrum and $R_{k}$ is the correlation matrix of the *k*th layer. Based on the output of the *k*th layer, each spectral vector of the next layer is updated, expressed as follows:

(5) $$x_{n}^{k+1}=q\left(y_{n}^{k}\right)x_{n}^{k}$$

where $q\left(y_{n}^{k}\right)$ is a nonlinear suppression function. The HSI spectral matrix of the next layer, which is applied to reconstruct a new CEM detector, will be updated continuously with the output of the current layer until the background pixels are entirely suppressed.

In ADHBS [38], the angular distance between the tested spectral vector and the known target spectrum, represented by θ, is used as the metric to separate the target and background pixels. The tested spectral vector gradually moves to the direction perpendicular to the target spectrum according to the metric θ in the spectral space. The angle distance in the *k*th layer is calculated by:

(6) $$\theta_{n}^{k}=\arccos\left\{\frac{\left|{\left(x_{n}^{k}\right)}^{T}\cdot d^{k}\right|}{{\left\|x_{n}^{k}\right\|}_{2}\cdot {\left\|d^{k}\right\|}_{2}}\right\}$$

where |•| means to take absolute value to ensure that the output angle is between 0 degrees and 90 degrees, and ${\left\|•\right\|}_{2}$ is to calculate the Euclidean norm of the vector.

Each spectral vector of the (k+1)th layer is adjusted by the following function:

(7) $$x_{n}^{k+1}=(1-\alpha_{n}^{k})x_{n}^{k}+\alpha_{n}^{k}d^{⊥}$$

where $d^{⊥}$ is an orthogonal vector of **d** and $\alpha_{n}^{k}$ determines the adjustment degree of the tested spectrum on the (k+1)th layer. The value of *α* is obtained by one power control function expressed as follows:

(8) $$\alpha_{n}^{k}={\left[\theta_{n}^{k}/90\right]}^{p}$$

where *p* controls the change rate of $\alpha_{n}^{k}$ which further controls the rate of the tested spectra moving to $d^{⊥}$. The change varies considerably from pixel to pixel, which is the key to distinguish target pixels and background pixels. The size of θ corresponding to background pixels is larger than that of the target pixels, so the movement rates of background spectra are faster than those of target spectra, which eventually makes the target pixels separated from the background clutter.

## 3. Proposed Method

### 3.1. Algorithm Flow Description

Inspired by related work on the hierarchical structure, we realize that the spectra transformation is beneficial to the separation of target and background, which results in better detection results. For MF, the information about the relationship between the target and the background is reflected in the output at a rough degree. The output value of the target pixels is greater than that of background pixels. In this paper, we utilize the output value of the MF detector to suppress the background spectra through a hierarchical structure in order to obtain one more accurate detection result. Each layer has one MF detector and the corresponding input and output, and the output of the current layer is used to adjust the input of the next layer. At the beginning of the next layer, the HSI input spectrum is multiplied by a constraint when one pixel is judged as a background pixel based on the output in the previous layer. In the whole iterative process, the output of each layer is the concrete embodiment of the convergence trend. In a word, the gradual convergence of backgrounds in the output eventually strips off the background clutter and retains the target pixels.

**Figure 1:**
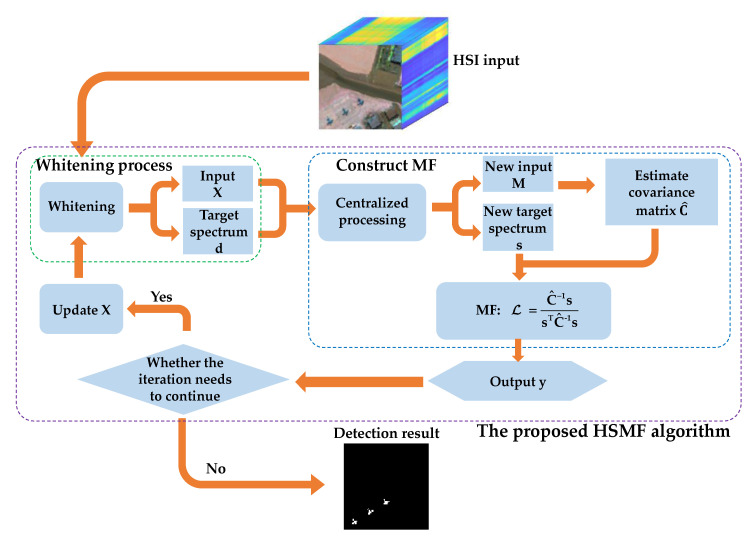
Flowchart of the proposed hierarchical background suppression on the matched filter detector (HSMF).

We determine the whole algorithm flow shown in Figure 1, and the proposed method is summarized as the following steps:

Step 1: Obtain the HSI spectral matrix and the target spectrum vector, then whiten both the HSI input and the prior target spectrum.
Step 2: Construct the MF detector for the current layer.
Step 3: Obtain the output and calculate the mean value of the output.
Step 4: Determine whether to continue the iteration. If the termination condition is met, go to step 5; otherwise, update the spectral input matrix of the next layer, and return to step 1.
Step 5: Output the final detection result.

### 3.2. Hierarchical Background Suppression on the MF Detector (HSMF)

Because the detector of each layer is developed from the traditional MF, the statistical models of the target pixels and the background clutter are similarly assumed to obey Gaussian distribution. In the proposed algorithm, we consider an observation spectral vector **x** (B×1) under this assumption with the following distribution:

(9) $$x∼N(as,\sigma^{2}\;\hat{C})$$

where $\;\hat{C}=\left(1/N\right)XX^{T}$ is the estimate covariance matrix of the background clutter, **s** is a (B×1) mean vector, and a and $\sigma^{2}$ are both nonnegative numbers which determine the mean value and the value of the covariance matrix, respectively.

**Figure 2:**
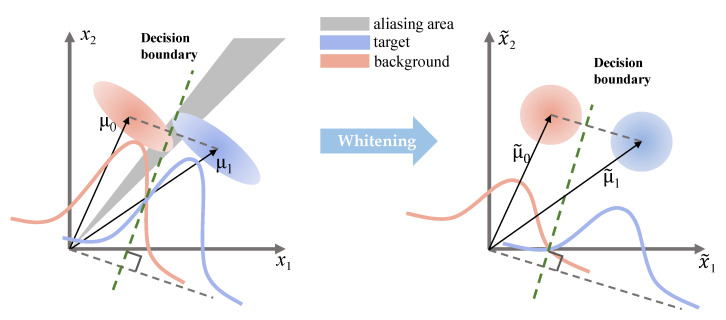
The original spectral observation space is shown at the left, and the whitened space with spherical covariance, created by the whitening transformation, is shown at the right. $\mu_{0}$ and $\mu_{1}$ represent the background and the target in the original spectral observation space, respectively. ${\;\tilde{\mu }}_{0}$ and ${\;\tilde{\mu }}_{1}$ represent the background and the target in whitened space, respectively. There is an aliasing area in the original spectral observation space. Both the target and background are considered as Gaussian distribution. The green dotted line works as a decision boundary.

Generally, the distribution of raw HSI data is severely discretized. There is considerable overlap between the distribution of the target and background in the original spectral observation space. By adopting the data whitening process, the data tend to a circular distribution instead of an elliptic distribution. The diagram of the whitening transformation is shown in Figure 2. As a result, the aliasing area between the target and background is reduced, and at the same time, it enlarges the spectral difference between the target and background pixels. When constructing the MF detector of different layers, not only does the HSI spectral matrix need whitening, but the vector **d** (B×1) containing the prior information is also whitened to be consistent with the input data. According to the known distribution characteristics, consider the whitening transformation:

(10) $$M={\;\hat{C}}^{-1/2}X,\;s={\;\hat{C}}^{-1/2}d$$

where the new spectral matrix and the new target spectrum vector are denoted by capital letters **M** and **s**, respectively. In the different layers of the iteration, the HSI spectral matrix suppresses its background components on the basis of the previous layer so that a better MF detector can be constructed to obtain more accurate detection results than the upper layer.

After the whitening process, the target pixels display the obvious separability to the background clutter. Then, according to the structure of the traditional MF detector, **M** and **s** in the whitened space are further centralized. That is, s subtracts a *B*×1 vector consisting of the average value of all pixels in each band, and M subtracts a *B*×*N* matrix obtained by copying the *B*×1 column vector.

Next, the preprocessed data are further used to construct the MF detector in different layers. The lowercase letter *k* indicates the number of iterations (layers). For the *k*th layer, the output of the HSMF is given by:

(11) $$y^{k}=\frac{s_{k}^{T}{\hat{C}}_{k}^{-1}}{s_{k}^{T}{\hat{C}}_{k}^{-1}s_{k}}M^{k}$$

where $s_{k}$ and ${\hat{C}}_{k}^{}$ define the filter coefficients with constraint (the new target spectrum vector) and the estimated covariance matrix of the background clutter for the *k*th layer, respectively.

Then, the next layer’s spectral input vector $x_{n}^{k+1}$ gets a corresponding update. By multiplying a constraint, the equation is formulated as follows:

(12) $$x_{n}^{k+1}=\lambda \left(y_{n}^{k}\right)x_{n}^{k}$$

where $\lambda \left(y_{n}^{k}\right)$ is an energy constraint function, and the constraint function is imposed on the raw input X, rather than the whitened **M**. In other words, the energy constraint function outputs a score to adjust the property of each pixel. When the score meets the suppression criterion, the tested pixel will be judged as a background pixel that needs to be suppressed in the next iteration.

With the update of the input, the MF detector of the next layer will be reconstructed. Herein, the energy constraint function is represented as

(13) $$\lambda \left(y_{n}^{k}\right)=\{\begin{array}{cc} 1, & y_{n}^{k}\geq \frac{1}{N}\sum_{n=1}^{N}y_{n}^{k}\\ \beta , & y_{n}^{k}<\frac{1}{N}\sum_{n=1}^{N}y_{n}^{k} \end{array}$$

where *β* is a parameter to constrain the backgrounds. *β* generally uses a positive number that is approximately zero. Such a choice is to make sure that the reconstructed estimate covariance matrix $\hat{C}$ is not singular. On the other hand, the purpose of the constraint is to decrease the influence of background pixels on constructing the MF detector in the next layer, and the output of the upper layer determines these background pixels. Therefore, weakening the influence of background pixels on the new MF detector by multiplying the constraint value *β* helps to improve the target detection performance of the next layer.

Typically, the output values of the MF detector objectively reflect the similarity between each HSI input spectrum and the prior target spectrum. Hence, $y_{n}^{k}$ corresponding to the target pixels is larger than $y_{n}^{k}$ corresponding to the background pixels. As the iteration continues, the number of background pixels detected increases. Therefore, with the support of the energy constraint function in different layers, we choose the mean of the score for each layer to determine whether the iteration needs to continue, as follows:

(14) $$\eta_{k}=\frac{1}{N}\sum_{n=1}^{N}\lambda \left(y_{n}^{k}\right)\overset{continue}{\underset{break}{≷}}\varepsilon$$

where *ε* is a threshold value. The detailed procedures of the proposed method are exhibited in Algorithm 1.

### 3.3. Theoretical Proof

Here, we prove that the mean value of a single layer output is suitable as a suppression criterion. We denote the MF detector of the *k*th layer as a vector ${ℒ}^{k}={\left(l_{1}^{k},l_{2}^{k},\ldots ,l_{B}^{k}\right)}^{T}$. The mean value of the *k*th layer ${\bar{y}}^{k}$ can be divided into targets and backgrounds:

(15) $$\begin{array}{cc} {\bar{y}}^{k}= & \frac{1}{N}\sum_{n=1}^{N}y_{n}^{k}\\ & =\frac{1}{N}\sum_{n=1}^{N}{ℒ}^{kT}M^{k}\\ & =\frac{1}{N}\left(\sum_{n=1}^{\tilde{N}}{ℒ}^{kT}{\tilde{M}}^{k}+\sum_{n=1}^{N^{\prime }}{ℒ}^{kT}M^{\prime }{}^{k}\right)\\ & =\frac{1}{N}\sum_{n=1}^{\tilde{N}}{ℒ}^{kT}{\tilde{M}}^{k}+\theta \end{array}$$

where $\tilde{N}$ and $N^{\prime }$ are the number of the background and target pixels, respectively, $\tilde{M}$ and ${Μ}^{\prime }$ are the spectral input of the background and target pixels, respectively, and θ is a constant representing the mean value of the output after the background pixels are completely suppressed. From Equations (12) and (15), the known background pixels are suppressed according to the output of the previous layer in the next layer. Now, we try to obtain the minimum value of the output mean value:

(16) $$\begin{array}{l} \underset{k\to k_{0}}{\lim}\frac{1}{N}\sum_{n=1}^{\tilde{N}}{ℒ}_{}^{kT}{\tilde{Μ}}^{k}=0\\ \underset{k\to k_{0}}{\lim}{\bar{y}}^{k}=\theta \end{array}$$

where $k_{0}$ represents a sufficiently large number of layers so that the background pixels are fully suppressed. The mean value of the output for each layer gradually converges to a constant θ. In addition, when the mean value of the output eventually converges to a constant, the proportion of the suppressed background pixels will increase, which causes the output of Equation (14) to become smaller. Thus, we set the threshold to determine whether the iteration is stopped, as shown in Equation (14).

The following proof analyzes the convergence of the output energy. Here, the MF detector as a vector is expressed in detail as follows:

(17) $${ℒ}^{k}=\frac{{\hat{C}}_{k}^{-1}\left(d^{k}-u^{k}\right)}{{\left(d^{k}-u^{k}\right)}^{T}{\hat{C}}_{k}^{-1}\left(d^{k}-u^{k}\right)}=\frac{{\hat{C}}_{k}^{-1}s_{k}}{s_{k}^{T}{\hat{C}}_{k}^{-1}s_{k}}$$

where the vector **u** represents the average value of all pixel values in each band. Then, we calculate the energy of the *k*th layer and the (k+1)th layer as follows:

(18) $${\left\|y^{k}\right\|}_{2}^{2}={\left\|{ℒ}^{kT}{Μ}^{k}\right\|}_{2}^{2}={\left\|\frac{s_{k}^{T}{\hat{C}}_{k}^{-1}{Μ}^{k}}{s_{k}^{T}{\hat{C}}_{k}^{-1}s_{k}}\right\|}_{2}^{2}=\frac{N}{s_{k}^{T}{\hat{C}}_{k}^{-1}s_{k}}$$

(19) $${\left\|y^{k+1}\right\|}_{2}^{2}={\left\|{ℒ}^{\left(k+1\right)T}M^{k+1}\right\|}_{2}^{2}={\left\|\frac{s_{k+1}^{T}{\hat{C}}_{k+1}^{-1}{Μ}^{k+1}}{s_{k+1}^{T}{\hat{C}}_{k+1}^{-1}s_{k+1}^{}}\right\|}_{2}^{2}=\frac{N}{s_{k+1}^{T}{\hat{C}}_{k+1}^{-1}s_{k+1}^{}}$$

Then, we need to compare the size of the two denominators [37]. Firstly, the difference between the covariance matrix of adjacent layers is calculated:

(20) $$\begin{array}{cc} {\hat{C}}_{k+1}^{}-{\hat{C}}_{k}^{} & =\sum_{1}^{N}\frac{1}{N}\left(\left(x_{n}^{k+1}-\mu^{k+1}\right){\left(x_{n}^{k+1}-\mu^{k+1}\right)}^{T}-\left(x_{n}^{k}-\mu_{x}^{k}\right){\left(x_{n}^{k}-\mu_{x}^{k}\right)}^{T}\right)\\ & =\sum_{1}^{N}\frac{1}{N}\left(\lambda^{2}m_{n}^{kT}m_{n}^{kT}-m_{n}^{k}m_{n}^{kT}\right)\\ & =WW^{T} \end{array}$$

where $W=\left[w_{1},w_{2},\ldots ,w_{N}\right]\in {\mathbb{R}}^{B\times N}$. According to the Sherman–Morrison formula [40], we suppose that ${\hat{C}}_{k}$ is an invertible square matrix and $\left({\hat{C}}_{k}+w_{n}w_{n}^{T}\right)$ is invertible if and only if $1+w_{n}^{T}{\hat{C}}_{k}^{-1}w_{n}\neq 0$. On this condition, we obtain

(21) $${\left({\hat{C}}_{k+1}+w_{n}w_{n}^{T}\right)}^{-1}={\hat{C}}_{k+1}^{-1}-\frac{{\hat{C}}_{k+1}^{-1}w_{n}w_{n}^{T}{\hat{C}}_{k+1}^{-1}}{1+w_{n}^{T}{\hat{C}}_{k+1}^{-1}w_{n}}$$

Then, we obtain the difference between the inverse covariance matrix of adjacent layers:

(22) $$\begin{array}{cc} {\hat{C}}_{k+1}^{-1}-{\hat{C}}_{k}^{-1} & ={\hat{C}}_{k+1}^{-1}-{\left({\hat{C}}_{k+1}+WW^{T}\right)}^{-1}\\ & =\sum_{n=1}^{N}\frac{{\hat{C}}_{k+1}^{-1}w_{n}w_{n}^{T}{\hat{C}}_{k+1}^{-1}}{1+w_{n}^{T}{\hat{C}}_{k+1}^{-1}w_{n}} \end{array}$$

Next, we obtain from Equations (20) and (22):

(23) $$\begin{array}{cc} s_{k}^{T}\left({\hat{C}}_{k+1}^{-1}-{\hat{C}}_{k}^{-1}\right)s_{k} & =\sum_{n=1}^{N}\frac{s_{k}^{T}{\hat{C}}_{k+1}^{-1}w_{n}w_{n}^{T}{\hat{C}}_{k+1}^{-1}s_{k}}{1+w_{n}^{T}{\hat{C}}_{k+1}^{-1}w_{n}}\\ & =\sum_{n=1}^{N}\frac{{\left\|s_{k}^{T}{\hat{C}}_{k+1}^{-1}w_{n}\right\|}_{2}^{2}}{1+w_{n}^{T}{\hat{C}}_{k+1}^{-1}w_{n}} \end{array}$$

where ${\left\|s_{k}^{T}{\hat{C}}_{k+1}^{-1}w_{n}\right\|}_{2}^{2}\geq 0$ and $w_{n}^{T}{\hat{C}}_{k+1}^{-1}w_{n}\geq 0$. We have the following size relationship:

(24) $$\frac{1}{s_{k}^{T}{\hat{C}}_{k+1}^{-1}s_{k}}\leq \frac{1}{s_{k}^{T}{\hat{C}}_{k}^{-1}s_{k}}$$

where $s_{k}^{T}={\left(d-u_{n}^{k}\right)}^{T}$. With the detected background pixels constrained, we have:

(25) $${\left\|s_{k}^{T}\right\|}_{2}^{2}\leq {\left\|s_{k+1}^{T}\right\|}_{2}^{2}$$

(26) $$\frac{1}{s_{k+1}^{T}{\hat{C}}_{k+1}^{-1}s_{k+1}}\leq \frac{1}{s_{k}^{T}{\hat{C}}_{k}^{-1}s_{k}}$$

Finally, we compare the output energy of adjacent layers:

(27) $$\frac{1}{N}{\left\|y^{k+1}\right\|}_{2}^{2}\leq \frac{1}{N}{\left\|y^{k}\right\|}_{2}^{2}$$

The above proof suggests that the output energy still converges, although $s_{k}$ and ${\hat{C}}_{k}^{-1}$ keep changing as the HSI spectral matrix is updated. In fact, these backgrounds suppressed in the upper layer have less impact on constructing the next layer detector, and the new detector pays more attention to the unsuppressed background.

Algorithm 1 describes the detailed procedures of the proposed method.
**Algorithm 1:** HSMF Algorithm**Input:**1. spectral matrix $X=\left[x_{1},x_{2},\cdots ,x_{N}\right]\in {\mathbb{R}}^{B\times N}$, target spectrum $d\in {\mathbb{R}}^{B\times 1}$, set threshold *ε*,**Initialization:**2.$\;k=1,\;X^{1}=X,\;n=1,2,\cdots ,N$, i=1,2,⋯,B,**Hierarchical Suppression:**3.$\;{\hat{C}}_{k}^{}=\left(1/N\right)X^{k}X^{kT}$,4.$\;M^{k}={\hat{C}}_{k}^{-1/2}X^{k},d^{k}={\hat{C}}_{k}^{-1/2}d^{k}$,5.$\;\mathrm{for}\;\mathrm{band}\;i:u_{i}^{k}=\left(1/N\right)\sum_{n=1}^{N}x_{n}^{k}$,6.$\;u_{}^{k}\in {\mathbb{R}}^{B\times 1}\overset{\mathrm{copy}\;\mathrm{column}\;\mathrm{vector}}{\to }U^{k}\in {\mathbb{R}}^{B\times N}$,7.$\;M^{k}=X^{k}-U^{k},s^{k}=d^{k}-u^{k}$,8.$\;{\hat{C}}_{k}^{}=\left(1/N\right){Μ}^{k}{Μ}^{kT}$,9.$\;y^{k}=\left(\left(s_{k}^{T}{\hat{C}}_{k}^{-1}\right)/s_{k}^{T}{\hat{C}}_{k}^{-1}s_{k}\right)M^{k}$,10.$\;x_{n}^{k+1}=\lambda \left(y_{n}^{k}\right)\times x_{n}^{k}$,11. k=k+1,**Stop Iteration:**12. $\eta_{k}=\frac{1}{N}\sum_{n=1}^{N}\lambda \left(y_{n}^{k}\right)$, if $\eta_{k}>\varepsilon$, come back to step 3; else, go to step 13,**Output:**13. final outputs: $y^{k}=\left[y_{1}^{k},y_{2}^{k},\cdots y_{N}^{k}\right]\in {\mathbb{R}}^{1\times N}$.

## 4. Experiment

In this section, three different experiments on classical HSI datasets are implemented to verify the effectiveness of HSMF. Here, we compare the proposed algorithms with several methods: SAM, CEM, CEM-IC, SMF, ACE, STD, MSD, SRBBH, hCEM, and ADHBS. Among them, SAM, CEM, SMF, and ACE are classical single-layer algorithms. In the following experiments, we set the spectral signature of the target based on the mean of all target samples of certain material in HSI, which is also adopted in [37,38]. The prior target spectral information of most detection algorithms is consistent so as to ensure fairness of the comparative experiments, except for MSD, STD, and SRBBH. For STD and SRBBH, instead of a feature vector, their prior information is expressed in the form of a matrix by a linear combination of a few atoms from the spectral dictionary. For MSD, the change of the spectral feature vectors is limited in a subspace of the band space.

In the experiment, we set a series of reasonable parameters for algorithms. Single-layer detectors such as SAM, CEM, SMF, and ACE do not need to preset any parameters. For MSD, SRBBH, and STD, the dual window size depends on the size of the target. Generally speaking, the inner window is larger than the target, and the outer window is larger than the inner window. To ensure that three methods have satisfactory results in three datasets, we set the sizes of their inner and outer windows to 13 and 19, 23 and 31, 13 and 19, respectively. For CEM-IC and hCEM, the original codes provided by previous studies are employed with default parameters. For ADHBS, the power function parameter is set to 3, which greatly reduces the number of iterations, and at the same time, it also obtains excellent detection results. For HSMF, we set suppression coefficient *β* = 0.0001 and threshold value *ε* = 0.01. The setting of these two parameters is analyzed in Section 5.3 in detail.

The experimental results of different detection algorithms are compared against the groundtruth. To visually illustrate the performance difference between our method and other algorithms, we plot results using receiver operating characteristic (ROC) [41,42]. ROC curves describe the dynamic relationship between the probability of detection (PD) and the false alarm rate (FAR), which is controlled by different thresholds. These two expressions are obtained as follows:

(28) $$PD=\frac{N_{c}}{N_{t}},FAR=\frac{N_{f}}{N_{b}}$$

where $N_{c}$, $N_{t}$ represent the number of correct target pixels detected and the total true target pixels in HSI, respectively, and $N_{f}$, $N_{b}$ represent the number of false alarm pixels and total number of background pixels, respectively. In addition, area under curve (AUC), whose range is between 0.5 and 1, also acts as a performance measure. Moreover, we compare the normalized AUC at lower FAR to exhibit the performance difference between different algorithms. The normalized AUC at lower FAR is obtained by normalizing AUC where FAR ranges from 0 to 0.001. Moreover, we plot the separability map to visually describe the separation of the target and background pixels for different detectors.

Regarding the experimental part of output energy convergence and mean convergence, we discuss this in Section 5.1 of the paper. The simulation software in the experiments is MATLAB 2018b, and the equipment used in all aforementioned methods is on an AMD 2600 personal computer with 16 GB RAM.

### 4.1. Experiment on San Diego Airport Data

**Figure 3:**
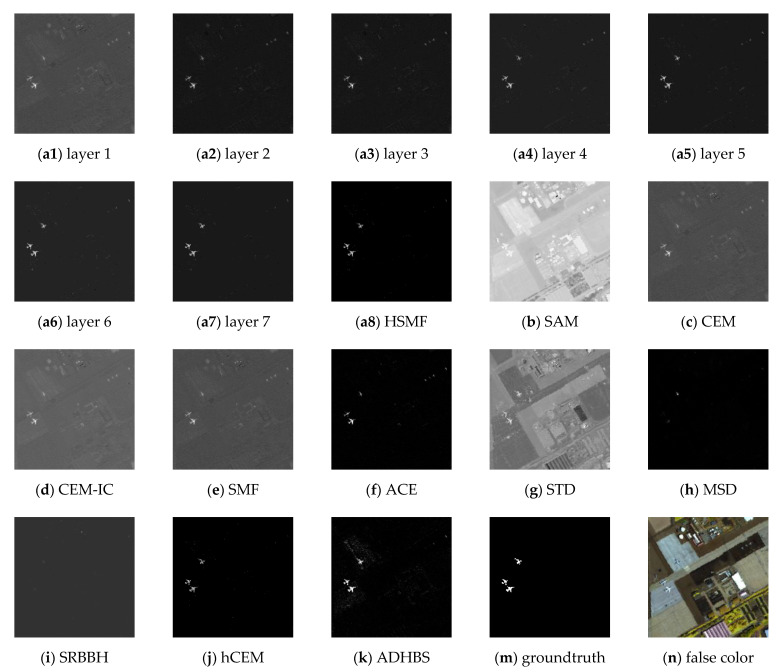
Detection results of different detectors on the San Diego Airport data. (**a1**–**a7**): results of HSMF for the first to the seventh layers. (**a8**): The final result of HSMF. (**b**–**k**): Results of spectral angle mapper (SAM), constrained energy minimization (CEM), inequality constraint on CEM (CEM-IC), spectral matched filter (SMF), adaptive coherence estimator (ACE), sparsity-based target detector (STD), matched subspace detector (MSD), sparse representation-based binary hypothesis (SRBBH), hierarchical CEM (hCEM), and angle distance-based hierarchical background separation (ADHBS). (**m**): The groundtruth information. (**n**) The false color image (bands 11, 40, 88).

**Figure 4:**
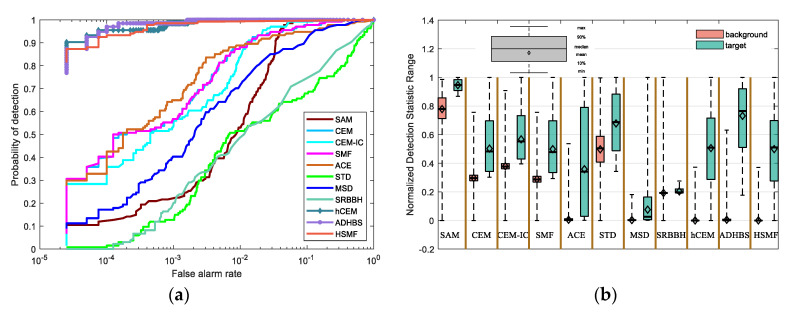
(**a**) The receiver operating characteristic (ROC) curves of different detectors on the San Diego Airport data. (**b**) The separability map of all detectors for the San Diego Airport data.

This classical dataset is collected by the Airborne Visible/Infrared Imaging Spectrometer (AVIRIS) [37,38] from the airport area in San Diego, USA, where the AVIRIS is a hyperspectral instrument designed, built, and operated by National Aeronautics and Space Administration (NASA). The raw spectral data consist of 224 spectral reflectance bands, which span from 0.4 to 2.5 μm. There are 200 × 200 pixels in each band, and the band number of the real hyperspectral image amounts to 189, where the low signal-to-noise ratio (SNR) and water absorption bands have been removed. The groundtruth and the false color image are shown in Figure 3m,n, respectively. The detection results of different detectors are shown in Figure 3, where Figure 3(a1–a7) show the detection result of different layers of HSMF. The ROC curves and the separability map are exhibited in Figure 4. As listed in Table 1, we compare both AUC values and AUC values at lower FAR of all detectors to describe the hyperspectral target detection performance in detail.

The iteration of HSMF stops in the 7th layer, and the final detection results are obtained. Combining the ROC curves with the AUC values in Table 1, we notice that three algorithms using hierarchical structure have better output results than other classical detectors. Comparing the AUC values of hCEM, ADHBS, and HSMF, although the difference between the three detectors is small, we can still find that HSMF has certain advantages. The AUC value of HSMF is second only to that of ADHBS, and the AUC value at lower FAR of HSMF is the maximum compared with the other detectors. From Figure 4b, we can further evaluate the separability between the target and background pixels. For ADHBS, the performance of separation is slightly better than HSMF and hCEM. For HSMF, the backgrounds are fully compressed, and the backgrounds and the targets are sufficiently separated. For SAM, CEM, CEM-IC, SMF, and ACE, the gaps between the backgrounds and the targets are small. For STD, MSD, and SRBBH, the area of the target box is partially overlapped with the background, which illustrates poor separability.

### 4.2. Experiment on Airport-Beach-Urban Data

**Figure 5:**
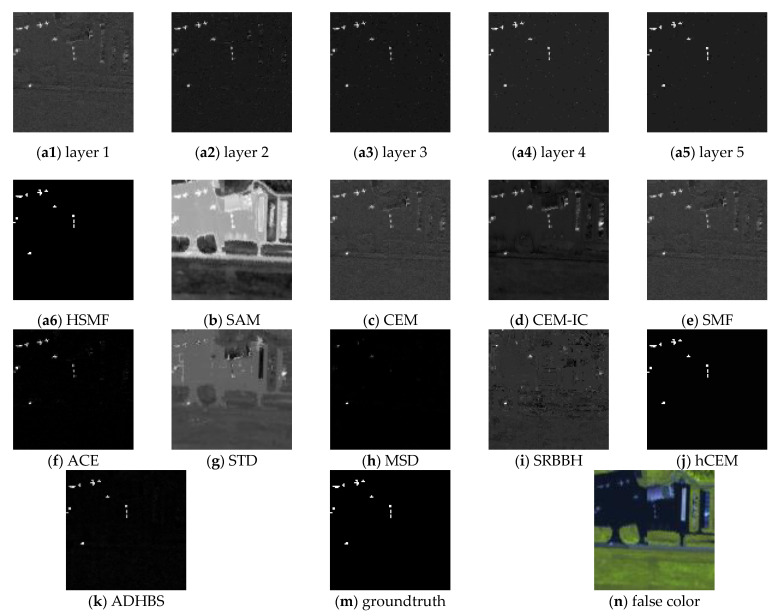
Detection results of the Airport-Beach-Urban data. (**a1**–**a5**): Results of HSMF for the first to the fifth layers. (**a6**): The final result of HSMF. (**b**–**k**): Results of SAM, CEM, CEM-IC, SMF, ACE, STD, MSD, SRBBH, hCEM, and ADHBS. (**m**): The groundtruth information. (**n**) The false color image (bands 23, 56, 72).

**Figure 6:**
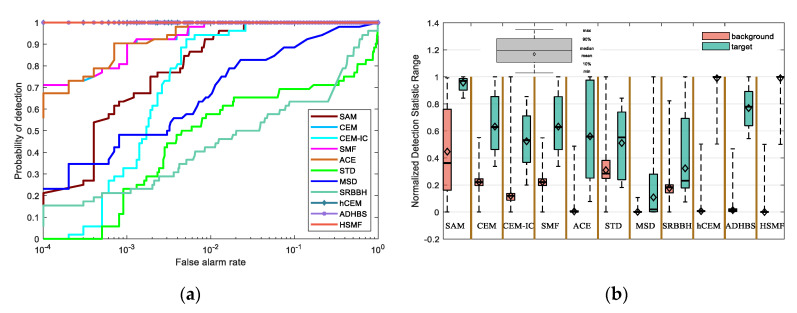
(**a**) ROC curves of different detectors on the Airport-Beach-Urban data. (**b**) The separability map of all detectors for the Airport-Beach-Urban data.

The Airport-Beach-Urban dataset is composed of multiple sets of sample images. The original images are collected by the AVIRIS and divided into size 100 × 100 manually. In addition, the spectral data retain 191 spectral reflectance bands after removing the noisy bands [43]. The groundtruth is labeled using the Environment for Visualizing Images (ENVI) software. The above-processed data are provided by [44]. In the experiment, we choose a sample image from the urban scenes in Gainesville, Florida, USA. The captured place of the image is Gainesville, and the spatial resolution is 3.5m. The spectral reflectance bands cover 0.4–2.5 μm. The groundtruth and the false color image are shown in Figure 5m,n, respectively. The detection results of different detectors are shown in Figure 5, where Figure 5(a1–a5) show the detection results of different layers of HSMF. The ROC curves and the separability map are exhibited in Figure 6. In Table 2, we record both AUC values and AUC values at lower FAR of all detectors to fully describe the performance of target detection.

For this dataset, the iteration in HSMF stops in the 5th layer. In Figure 6, the ROC curves of hCEM, ADHBS, and HSMF are at the top of the image, indicating a better detection result. Similarly, the AUC values and the AUC values at lower FAR show that HSMF is better than classical detectors. According to the separability map in Figure 6b, we compare the separation of the targets and the backgrounds in all algorithm results. For HSMF, the gap between the backgrounds and the targets is the largest. For hCEM, the separability is greatly close to HSMF. For CEM, CEM-IC, SMF, ACE, STD, and ADHBS, the targets and the backgrounds have no overlap, but the gaps are small. For SAM, the area of the target box is larger than the others, which demonstrates that the background is not sufficiently suppressed. For MSD, STD, and SRBBH, they have a bad separability because the area of the target boxes is partially or completely overlapped with that of the background boxes.

### 4.3. Experiment on Urban Data

**Figure 7:**
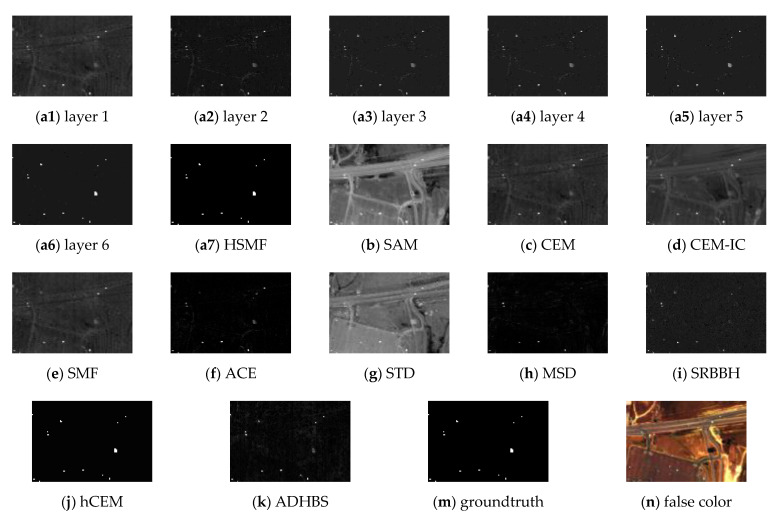
Detection results of different algorithms on the Urban data. (**a1**–**a6**): Results of HSMF for the first to the sixth layers. (**a7**): The final result of HSMF. (**b**–**k**): Results of SAM, CEM, CEM-IC, SMF, ACE, STD, MSD, SRBBH, hCEM, and ADHBS. (**m**): The groundtruth information. (**n**) The false color image (bands 7, 48, 144).

**Figure 8:**
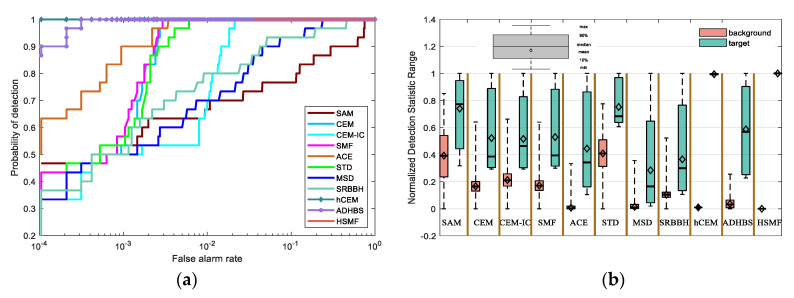
(**a**) ROC curves of different detectors on the Urban data. (**b**) The separability map of all detectors for the Urban data.

As one of the most widely used hyperspectral datasets, the Urban dataset is collected by the Hyperspectral Digital Imagery Collection Experiment (HYDICE) sensor over California, USA, where the HYDICE sensor is manufactured by Hughes Danbury Optical Systems. The original image scene consists of 307 × 307 pixels. The raw spectral data contain 210 spectral reflectance bands in the wavelength ranging from 0.4 to 2.5 μm. After removing the water-absorbed and noisy bands, the number of bands is lessened to 162 [45]. The groundtruth of the Urban scene has several classes, mainly including asphalt, grass, tree, and roof. In the experiment, we only choose a subset of 80 × 120 pixels in the upper left corner of the large image, where the roof is chosen as the groundtruth. The groundtruth and the false color image are shown in Figure 7m,n, respectively. The detection results of different detectors are shown in Figure 7, where Figure 7(a1–a6) show the detection results of different layers of HSMF. The ROC curves and the separability map are exhibited in Figure 8. In Table 3, we record both AUC values and AUC values at lower FAR of all detectors to describe the target detection performance exhaustively.

In this experiment, HSMF obtains the detection output after six layers of hierarchical suppression. Figure 8a shows that the ROC curves of hCEM and HSMF are on the top of the other detectors. In other words, hCEM and HSMF outperform other methods. For ADHBS, the AUC value at lower FAR is worse than hCEM and HSMF. In Figure 8b, the separability of hCEM and HSMF still behaves well. For SAM and STD, their backgrounds are not effectively suppressed. For the others, the gaps between the target boxes and the background boxes are relatively small.

## 5. Discussion

### 5.1. Energy Output Convergence and Mean Convergence

In this section, we test and verify the convergence performance of HSMF for different layers. Meanwhile, the rationality of the suppression criterion, chosen in HSMF, is discussed. In fact, the mean value of the absolute output value also gradually converges to a constant after multiple iterations. The constant is an ideal value when the background pixels are completely suppressed, and only the target pixels remain.

The square of the Euclidean norm of each layer output, expressed as ${\left\|y^{k}\right\|}_{2}^{2}$, works as the output energy of the corresponding layer. Curves drawn by the output energy of different layers exhibit the energy convergence trend.

**Figure 9:**
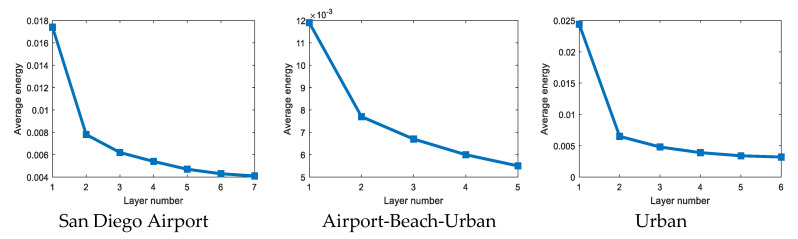
The average energy of HSMF with different layers on three hyperspectral data.

As shown in Figure 9, the output energy tends to converge after a few iterations on three hyperspectral images. The sharp drop in energy between the first and second layers is the most obvious. The convergence tends to be flat after the second layer. It is easy to find that the first two layers suppress most of the background pixels, and the remaining layers will suppress the background at a finer level to improve the detection accuracy.

**Figure 10:**
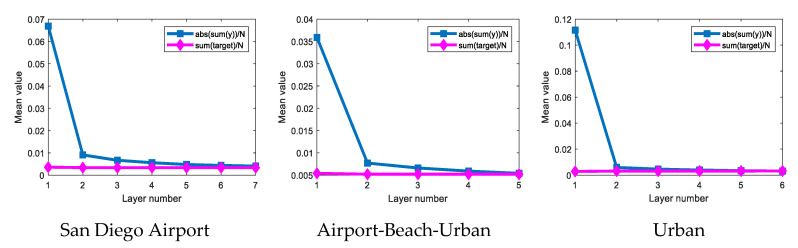
Output mean value of different layers on three hyperspectral data.

In Figure 10, the blue curves denote the mean value of the absolute output value, and the pink curves represent the mean value of only target-remained output where the background output is considered to be zero. The two output means can be expressed, respectively, as:

(29) $${\bar{y}}^{k}=\frac{1}{N}\sum_{1}^{N}\left|y_{n}^{k}\right|$$

(30) $${{\bar{y}}^{\prime }}^{k}=\frac{1}{N}\sum_{1}^{N^{\prime }}\left|{\tilde{y}}_{n}^{k}\right|$$

where ${\tilde{y}}_{n}^{k}$ represents the target pixels output, and $N^{\prime }$ denotes the number of target pixels. The pink curves fluctuate in a very small range. This is because the target pixels are well preserved. The blue curves converge quickly, and the sharp drop between the first and second layers is the most obvious. In line with the expectation, the blue curves gradually converge to the pink curves. In other words, the mean value of the absolute output value eventually equals the mean value of the only target-remained output, which accords with Equation (16) and means that the target pixels are preserved while the background pixels are suppressed.

Since the process of the background suppression is irreversible, once a pixel is detected as a background pixel, its input in the next layer will be suppressed. Hence, detection performance is determined by the different suppression criteria. Here, we choose several suppression criteria for comparison. Besides mean value, we also choose one-eighth mean, quarter mean, one-half mean, mean, and three-quarter mean.

**Figure 11:**
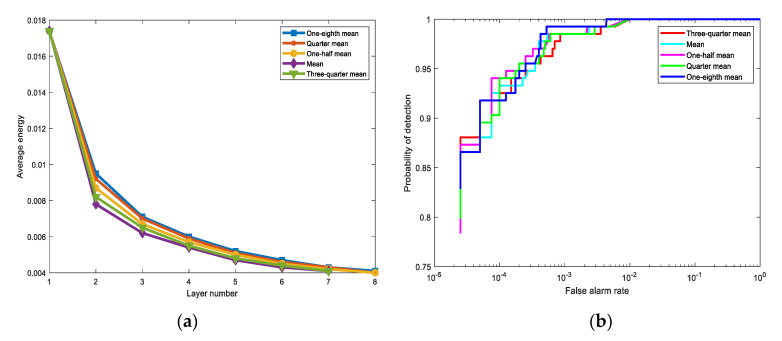
The effects of the different suppression criteria on the San Diego Airport data. The suppression criteria used in the comparative experiments are one-eighth mean, quarter mean, mean, one-half mean, and three-quarter mean. (**a**) The average output energy of different layers for different suppression criteria. (**b**) The ROC curves comparison for different suppression criteria.

As exhibited in Figure 11a, different suppression criteria require different iteration layers, and the average energy of each layer is different from each other. When the suppression criterion is the mean value and three-quarter mean, the number of layers is seven. For other suppression criteria, eight iterations are required. According to Figure 11b, although the ROC curves of different suppression criteria remain different, the detection results are relatively close. Combining Figure 11a,b, when the mean value works as the suppression criterion, the detection only requires fewer iterations while the detection performance is also excellent.

### 5.2. Time Performance

In Table 4, we list the running time of different detection algorithms on the three datasets. Meanwhile, we count the number of iterations and calculate the time consumption of each layer. Some detectors have similar time performance, and the other detectors take a long time to get the output.

For SAM, it only needs to calculate the angular distance between each pixel and the prior spectral information. Thus, its running time is the shortest on the three datasets. For CEM and SMF, they just design a single layer FIR filter and MF, respectively, which are both outstanding in time performance. For STD and SRBBH, both are based on the sparse representation, which often costs considerable time for recovering the sparse vectors. At the same time, it is necessary to take time to construct a local background dictionary and a local target dictionary. As the dual windows move, a tremendous amount of computation needs to be required. MSD is the worst in terms of time performance because it uses singular value decomposition to construct the target and background subspaces. For CEM-IC, it also takes relatively little time to output the detection results. For ADHBS, the number of iterations is the highest on the last two datasets, and the running time is also more than hCEM and HSMF on the three datasets. For the hCEM, the total running time is less than ADHBS and HSMF on three datasets. For HSMF, it costs slightly more time than hCEM, but the number of iterations is the lowest. It means that HSMF has the best convergence performance. Because the MF coefficients with constraint and the estimated covariance matrix need to be recalculated at each layer in the new whitened space, it costs the most time in each layer.

### 5.3. The Effect of Parameter

In the proposed method, we need to set two parameters, namely suppression coefficient *β* and threshold value *ε* manually. These two parameters play an important role in detection performance. The size of *β* controls the degree of background suppression, and the size of *ε* controls the stop condition. This section discusses the influence of different parameter values on the experiment, mainly including the number of iterations and detection performance.

**Figure 12:**
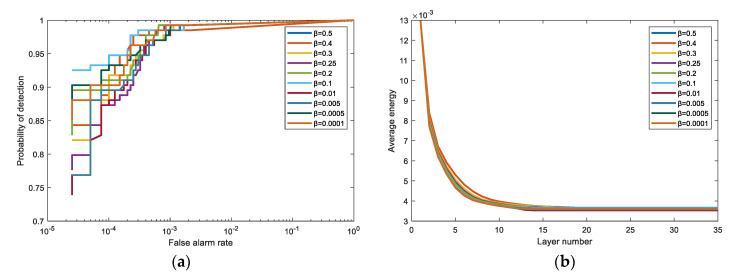
The effects of the different *β* values on the San Diego Airport data. We choose 10 different values for comparison, including 0.5, 0.4, 0.3, 0.25, 0.2, 0.1, 0.01, 0.005, 0.0005, and 0.0001. (**a**) The ROC curves comparison for different *β* values. (**b**) The average output energy of different layers for different *β* values.

According to Figure 12b, the average output energy of different *β* values is very close in the same layer, which means that the different *β* values have little effect on the average output energy. In other words, under the suppression criterion in this paper, the output energy convergence is less affected by the parameter *β.* However, the suppression coefficient *β* has a relatively large impact on the number of iterations required for final convergence and the detection performance (i.e., AUC). Combining Table 5 and Figure 12a, as the value decreases the number of layers as are necessary also drops to a constant. Until the *β* value is not greater than 0.1, the number of iterations tends to be stable. In addition, once the *β* value is between 0.5 and 0.4, the AUC is relatively small. When the *β* value is smaller than 0.4, AUC tends to be stable, while AUC at lower FAR fluctuates in a small range. When the *β* value is 0.1 or 0.0001, AUC at lower FAR has better results. On the Airport-Beach-Urban data and Urban data, the parameter *β* also shows this trend. Herein, we only discuss the San Diego Airport data.

**Figure 13:**
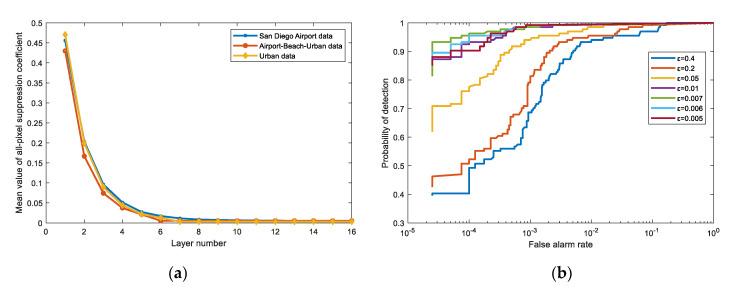
(**a**) The mean value of all pixels’ suppression coefficient in different layers on three different datasets. (**b**) The ROC curves comparison for different *ε* values where *ε* value contains 0.4, 0.2, 0.05, 0.01, 0.007, 0.006, and 0.005.

In the part of discussing the threshold value, we set *β* = 0.0001, which keeps up with the experiment in Section 4. In Figure 13a, the ordinate represents the mean value of all pixels’ suppression coefficient in different layers, as shown in Equation (14). For three different data, their convergence trends are the same, and they all tend to a minimal value in the end, which means that the selection of the threshold value must be greater than the convergence value. Otherwise, the threshold has no ability to constrain the process of the algorithm. In datasets Airport-Beach-Urban data and Urban, they only need six and seven iterations to converge to a constant. Hence, in order to better distinguish the impact of different thresholds on the detection performance, we use San Diego Airport data for comparison experiments.

Table 6 exhibits that as the threshold decreases, the number of iterations gradually increases until the threshold fails to terminate the iteration. With reference to the AUC value in Table 6 and the ROC curves in Figure 13b, the detection performance has a significant disadvantage when the threshold is set relatively large. From Table 6, we also find that the AUC at lower FAR has a trend where the value will increase first and then decrease. Considering the number of iterations and detection performance, we choose an appropriate threshold value, i.e., *ε* = 0.1, in the experiments.

In the parameter discussion, we conclude that the selection of parameters does not have an optimal value. It is crucial to choose reasonable parameters for different experiments. On the one hand, we must ensure the efficiency and accuracy of detection, and on the other hand, the parameters can be generalized to some extent.

## 6. Conclusions

In this paper, we are not limited to the single-layer detection of the MF detector but continuously construct a new MF detector for each iteration via the hierarchical structure to pursue better detection results. The mean output criterion adopted in the hierarchical background suppression also speeds up the output energy convergences, so the proposed method has fewer iterations compared with other hierarchical detectors. After the final iteration, the backgrounds are effectively suppressed while the targets are retained. Experimental results on three real hyperspectral images prove the outstanding detection performance of HSMF. From the ROC curves of the three datasets in the experimental part, it is straightforward to judge that HSMF has a better detection performance than classical algorithms. Compared with hCEM and ADHBS, HSMF maintains an excellent detection performance with the least number of iterations. 

## Figures and Tables

**Table 1:** Area under the receiver operating characteristic (ROC) curves (AUC) values and AUC values at lower false alarm rate (FAR) of all detectors for the San Diego Airport data.

**Algorithm**	SAM	CEM	CEM-IC	SMF	ACE	STD
**AUC**	0.9860	0.9905	0.9941	0.9904	0.9772	0.8441
**AUC (lower FAR)**	0.1768	0.4990	0.4485	0.5017	0.5406	0.0868
**Algorithm**	MSD	SRBBH	hCEM	ADHBS	HSMF	
**AUC**	0.9687	0.8649	0.9924	**0.9999** ^1^	0.9925	
**AUC (lower FAR)**	0.2903	0.0941	0.9562	0.9470	**0.9587** ^1^	

^1^ The bold represents the maximum.

**Table 2:** AUC values and AUC values at lower FAR of all detectors for the Airport-Beach-Urban data.

**Algorithm**	SAM	CEM	CEM-IC	SMF	ACE	STD
**AUC**	0.9972	0.9994	0.9968	0.9994	0.9995	0.7695
**AUC (lower FAR)**	0.4081	0.7564	0.1325	0.7585	0.7560	0.0321
**Algorithm**	MSD	SRBBH	hCEM	ADHBS	HSMF	
**AUC**	0.9503	0.7994	**1.0000**	**1.0000**	**1.0000**	
**AUC (lower FAR)**	0.3462	0.1572	**1.0000**	**1.0000**	**1.0000**	

The bold represents the maximum.

**Table 3:** AUC values and AUC values at lower FAR of all detectors for the Urban data.

**Algorithm**	SAM	CEM	CEM-IC	SMF	ACE	STD
**AUC**	0.9020	0.9990	0.9940	0.9991	0.9996	0.9988
**AUC (lower FAR)**	0.4778	0.4593	0.4296	0.4667	0.7259	0.4667
**Algorithm**	MSD	SRBBH	hCEM	ADHBS	HSMF	
**AUC**	0.9779	0.9719	**1.0000**	0.9998	**1.0000**	
**AUC (lower FAR)**	0.4519	0.4259	**1.0000**	0.8593	**1.0000**	

The bold represents the maximum.

**Table 4:** Time performances of different detectors on the three datasets (keep four decimal places in the units of seconds).

Algorithms	San Diego Airport		Airport-Beach-Urban		Urban	
	Total Time	Time/Layer	Total Time	Time/Layer	Total Time	Time/Layer
SAM	0.0576	0.0576	0.0249	0.0249	0.0177	0.0177
CEM	0.1659	0.1659	0.0402	0.0402	0.0278	0.0278
CEM_IC	0.4060	0.4060	0.2855	0.2855	0.2016	0.2016
SMF	0.2054	0.2054	0.0591	0.0591	0.0487	0.0487
ACE	0.5949	0.5949	0.0815	0.0815	0.0936	0.0936
STD	76.5828	76.5828	17.8557	17.8557	17.5028	17.5028
MSD	369.7743	369.7743	92.2054	92.2054	68.2234	68.2234
SRBBH	59.1558	59.1558	10.8585	10.8585	14.2643	14.2643
hCEM	3.8807(15) ^1^	0.2587	1.2482(8)	0.1560	1.1822(8)	0.1478
ADHBS	4.3435(11)	0.3949	2.6310(17)	0.1547	3.3069(22)	0.1503
HSMF	4.2831(7)	0.6119	1.3298(5)	0.2659	1.2975(6)	0.2163

^1^ The value in parentheses indicates the number of iterations.

**Table 5:** The number of iterations and detection performance for different *β* values.

**Suppression coefficient *β***	0.5	0.4	0.3	0.25	0.2
**AUC**	0.9851	0.9851	0.9925	0.9925	0.9925
**AUC (lower FAR)**	0.9638	0.9638	0.9476	0.9443	0.9592
**Number of iterations**	35	27	26	23	21
**Suppression coefficient *β***	0.1	0.01	0.005	0.0005	0.0001
**AUC**	0.9925	0.9925	0.9925	0.9925	0.9925
**AUC (lower FAR)**	0.9742	0.9310	0.9409	0.9562	0.9599
**Number of iterations**	16	16	16	16	16

**Table 6:** The number of iterations and detection performance for different *ε* values.

**Threshold value *ε***	0.4	0.2	0.05	0.01	0.007	0.006	0.005
**AUC**	0.9904	0.9906	0.9922	0.9925	0.9925	0.9925	0.9925
**AUC (lower FAR)**	0.5599	0.6359	0.8718	0.9587	0.9698	0.9670	0.9599
**Layer number**	1	2	4	7	9	11	NaN ^1^

^1^ NaN (Not a Number) indicates that the termination condition cannot be met, and the algorithm will iterate indefinitely.

## Data Availability

No new data were created or analyzed in this study. Data sharing is not applicable to this article.
